# Integrated care models for youth mental health: A systematic review and meta-analysis

**DOI:** 10.1177/00048674241256759

**Published:** 2024-06-07

**Authors:** Catherine McHugh, Nan Hu, Gabrielle Georgiou, Michael Hodgins, Sarah Leung, Mariyam Cadiri, Nicola Paul, Vikki Ryall, Debra Rickwood, Valsamma Eapen, Jackie Curtis, Raghu Lingam

**Affiliations:** 1 Mindgardens Neuroscience Network, Sydney, NSW, Australia; 2 Discipline of Psychiatry, University of New South Wales, Sydney, NSW, Australia; 3 Population Child Health Research, University of New South Wales, Sydney, NSW, Australia; 4 Headspace National Youth Mental Health Foundation, Melbourne, VIC, Australia; 5 Faculty of Health, University of Canberra, Canberra, ACT, Australia

**Keywords:** Youth, integrated care, coordinated care, depression

## Abstract

**Objectives:**

To evaluate the effectiveness of integrated models of mental healthcare in enhancing clinical outcomes, quality of life, satisfaction with care and health service delivery outcomes in young people aged 12–25 years. A secondary objective was to identify common components of integrated mental health interventions.

**Methods:**

A systematic review and meta-analysis of studies published 2001–2023 that assessed clinical or health service use outcomes of integrated care, relative to treatment as usual, for any mental health condition in 12–25 years old accessing community-based care.

**Results:**

Of 11,444 titles identified, 15 studies met inclusion criteria and 6 studies were entered in the meta-analysis. Pooled effect size found integrated care was associated with a greater reduction in depressive symptoms relative to treatment as usual at 4–6 months (standardised mean difference = −0.260, 95% confidence interval = [−0.39, −0.13], *p* = 0.001). Of the seven studies reporting access or engagement, all reported higher rates of both in the intervention arm. The most frequent components of integration were use of a multidisciplinary team (13/15 studies), shared treatment planning (11/15) and workforce training in the model (14/15).

**Conclusions:**

Integrated models of mental healthcare are associated with a small, but significant, increase in effectiveness for depressive symptoms relative to treatment as usual. Given integrated care may increase access and engagement, future research should focus on assessing the impact of integrated care in a wider range of settings and outcomes, including clinical and functional recovery, satisfaction with care and system-level outcomes such as cost-effectiveness.

## Background

Health services in many countries are facing an unprecedented demand for mental healthcare for young people (Hu et al., 2022; Kalb et al., 2019; Mercado et al., 2017; Perera et al., 2018). Reports have highlighted the significant gaps in access to timely assessment and evidence-based treatment, with sustained shortages of specialist workforces and inadequate training pathways. In the face of such demand, it can be challenging for health services to deliver high-quality mental healthcare which, according to World Health Organization (2016b), should be timely, effective and evidence-based, safe and person-centred. The delivery of high-quality mental healthcare to young people and their families faces additional challenges. Young people accessing care frequently describe difficulty in engaging with services that are not orientated towards young people and may be particularly vulnerable to geographical and financial barriers to accessing care (Burkhart et al., 2020; Reardon et al., 2017).

Youth mental health services are commonly divided by age (child/adolescent, adult), mental health condition (e.g. psychosis, mood disorder, drug and alcohol), setting of treatment (primary care, emergency department, hospital or community clinic based) and funding model (private or health insurer, governmental, non-government). At a system level, this fragmentation between services contributes to duplication and potentially unnecessary change in service providers, wasting of limited resources, missed opportunities for collaboration and a failure to deliver person-centred care (Western Australian Association for Mental Health, 2018). For consumers and carers, this can lead to confusion in their interactions with the mental health system, as well as dissatisfaction and disengagement with care (State of Victoria, 2021).

The delivery of integrated healthcare is one strategy to address the fragmentation of mental health services and care. Integrated healthcare models can aim to integrate different domains of healthcare (physical health, mental health, substance use) or governmental and social services with health (education, housing, welfare) termed horizontal integration, or different levels of healthcare (primary, secondary or tertiary and specialist care) termed vertical integration (World Health Organization, 2016a). Such integration has the potential to improve access to care, as well as quality of care, in an efficient and cost-effective manner.

Leading frameworks of integrated care describe the degree of integration occurring along a continuum from basic coordination between providers to highly integrated services with system integration (Hodgins et al., 2022). Mechanisms of integration may be conceptualised as operating at the level of the health system, the organisation or clinical service and/or within the intervention itself (World Health Organization, 2016a). System-level components of integration include those related to planning and governance, such as shared commissioning and funding models, the development of intersectoral or interagency partnerships and inclusion of research and evaluation (Bartholomeusz and Randell, 2022). These system-level factors are necessary precursors to the components of integration that act at the level of the clinical service, which will be the focus of this review.

Although integration of health care has been identified as a priority across many health systems, understanding the impact on service delivery and clinical outcomes is complex. Australia has been a global leader in developing integrated early intervention mental healthcare services for young people through the headspace model (Malla et al., 2016; Rickwood et al., 2019). Services have increased help-seeking and access to care in young people in distress and have been well received by young people and their families (Rickwood et al., 2015, 2017).

Integrated mental healthcare models for discrete cohorts of young people, e.g. those being treated for first episode psychosis, have reported a significant positive effect on clinical symptoms, quality of life and engagement with treatment (Fusar-Poli et al., 2017; Henry et al., 2010). In adults, delivery of integrated mental healthcare to treat depressive and anxiety disorder in primary care settings has been found to be clinically effective and cost-effective (Archer et al., 2012; Holst et al., 2018; Richards et al., 2016). The impacts of integrated care on a broader range of youth mental health conditions are less well understood. In a review of integrated youth mental health services for adolescents and young adults (12–25 years), Hetrick et al. (2017) reported symptomatic and functional recovery in the majority of young people, except for those with more severe presenting symptoms. However, these findings were largely drawn from formal evaluations of these services, which did not include control groups. A meta-analysis by Asarnow et al. (2015), which focused on behavioural health symptoms in children and young people aged 0–21 years, reported integrated care models were associated with a greater reduction in a symptoms relative to treatment as usual (TAU). The review included a high proportion of studies examining children (<12 years) receiving care for neurodevelopmental conditions such as attention-deficit hyperactivity disorder (ADHD) or non-specific behavioural symptoms, presentations that are vastly different in terms of aetiology and treatment required to those seen in youth mental health settings. It remains unclear as to how generalisable these findings are to cohorts of young people experiencing mental health symptoms. Outcomes such as engagement with care have not been examined in previous systematic reviews yet have been identified as particularly crucial to youth mental health service delivery (Fusar-Poli, 2019). Finally, there is a need for increased specificity as to the components of integrated care included in youth mental health interventions, in order to consider the potential impact in different health settings and systems.

Building and expanding on this previous work, the current systematic review aimed to: (1) evaluate the effectiveness of integrated models of youth mental healthcare on a broader range of outcomes, including both mental health outcomes, such as clinical symptoms, functioning and quality of life and health service outcomes, including access and satisfaction with care in young people aged 12–25 years and (2) identify the common components of integrated care models.

## Methods

### Search strategy

We conducted a systematic review and meta-analysis according to the Preferred Reporting Items for Systematic Reviews and Meta-Analyses guidelines (Moher et al., 2009). A search was conducted of peer-reviewed, English language research literature from January 2001 to December 2023 using PubMed, SCOPUS and PsycINFO databases. The project was not registered with PROSPERO prior to commencing, though the objectives, eligibility criteria and outcomes of interest were agreed upon by co-authors. Search terms are included in Supplemental Table 1. Studies that evaluated models of integrated mental healthcare for young people aged 12–25 years were included where they met the following inclusion criteria: (1) participants had been diagnosed with at least one mental health condition, including autism spectrum disorder and ADHD, (2) studies included an intervention and control group, (3) studies reported an outcome measure related to clinical symptoms, functioning or health service use and (4) studies were conducted in community-based settings, though may have focused on integrating community-based care with hospital-based care. Clinical services based in community settings are defined broadly to include any clinical care outside of the inpatient mental health system. This includes primary care, as well as more specialised mental health services.

Studies were excluded where participants’ primary diagnoses were related to alcohol or substance use disorders.

### Identification of studies

**Figure 1.**
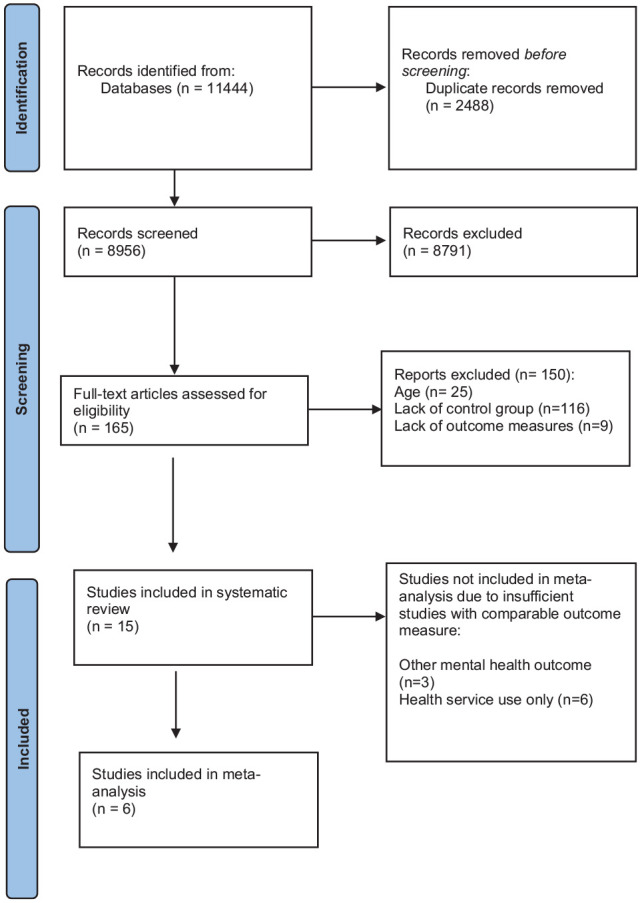
PRISMA flowchart. PRISMA: Preferred Reporting Items for Systematic Reviews and Meta-Analyses.

Over 11,444 titles or abstracts were screened for relevance. After screening, 165 sources were identified as potentially relevant and the abstract and full text of these were reviewed by two authors to determine eligibility (GG, CM). Fifteen studies met the inclusion criteria for this review (Figure 1).

### Data extraction and synthesis

**Figure 2.**
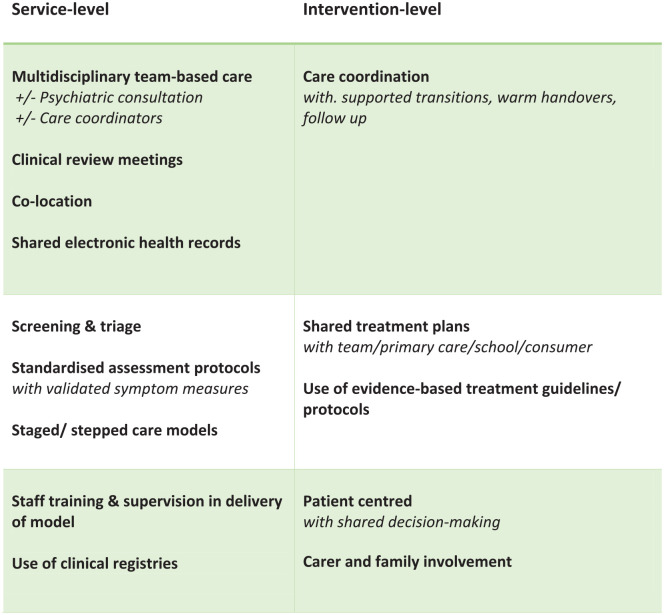
Components of integrated care models.

Data were systematically extracted from the included studies by two authors (CM, NH) using a pre-specified pro forma. Information extracted included study design, sample characteristics (sample size, age, gender, setting), intervention characteristics (duration, components of integration) and outcome measures. The components of integration were extracted using a model adapted from the Collaborative Care Model by Yonek et al. (2020). Two authors (CM, GG) further adapted these components and incorporated components from leading models of youth mental healthcare (see Figure 2; Hetrick et al., 2017; Rickwood et al., 2019). The core components as reported by Yonek et al. (2020) were factors that operate at the level of the clinical service and intervention itself. Additional components from youth mental health models of integration included patient-centred care (represented as including patient preference in treatment selection), family or carer involvement and aspects of team or clinical collaboration, including clinical review meetings and staff supervision and training in the model of integrated care.

Outcome measures of interest were clinical outcomes and health service outcomes. Clinical outcomes included measures of clinical symptoms, clinical improvement, functioning and/or quality of life. Health service outcomes included measures of attendance, engagement and patient satisfaction. Many studies reported outcome measures at several follow-up time-points; as all studies reported the outcome at a time-point between 4 and 6 months of follow-up, these outcomes were extracted for analysis.

### Assessment of study quality

The risk of bias of the included studies was evaluated using the Cochrane Collaboration’s tool for assessing risk of bias (Higgins et al., 2011).

### Meta-analysis

Post-intervention outcomes as means and standard deviations (SDs) adjusted for baseline differences were used for analysis. Standardised mean difference (SMD; equivalent to Cohen’s *d*) and standard error were estimated for each study using a random effects model in Comprehensive Meta-Analysis software (Egger et al., 1997). Between-study heterogeneity in effect sizes was examined using *I*-square and *Q*-statistics. Potential publication bias was assessed using a funnel plot and Egger’s regression (Egger et al., 1997).

## Results

### Study characteristics

Fifteen studies reporting 13 interventions met inclusion criteria for the systematic review, of which 11 studies were randomised controlled trials and 4 studies were observational (either case-control or retrospective cohort). The majority of studies (14 of 15) were conducted in urban settings and in the United States, aside from single studies conducted in Australia, Canada and Ireland. The majority of interventions focused on the integration of mental health services into primary care settings; i.e. vertical integration. Less common were studies focusing on horizontal integration, such as within the school system.

Ten studies sampled only young people with depression; five studies included young people with depression and/or other common youth mental health conditions including generalised anxiety disorder, social anxiety, psychotic-like experiences, co-morbid substance use, ADHD or disruptive disorders. Two studies included any young person referred for a mental health evaluation. Six studies excluded participants with recent suicidal ideation or behaviour or common co-morbidities, such as substance use disorder or bipolar disorder.

Nine studies examined the impact of integration on clinical outcomes (Table 1) and seven evaluated the health service use outcomes of integrated care (Table 2). Two studies examined both clinical outcomes and health service use.

**Table 1.** Summary of studies evaluating clinical impact of integrated YMH models.

Study	Participants, *n*		Sample and settling			Intervention			Outcome measures		
	Intervention	Control	Age (years)	Group	Setting	Time frame of intervention	Intervention	Control group	Primary	Other	Significance
Asarnow et al. (2005)	211	207	13–21	Depression	Paediatric primary care sites United States	6 months	Co-location, care coordination, use of evidence-based treatment protocols (± CBT ± medication)	PCP + additional training and materials	CES-D	SF-12, YSR-112 Patient satisfaction Access (number of MH visits)	Intervention associated with fewer depressive symptoms, higher MH-related QoL, satisfaction with care and access rates
Clarke et al. (2005)	75	77	12–18	Depression	Paediatric primary care United states	12 months	Co-location, use of treatment protocol (SSRI + brief CBT: cognitive restructuring or behavioural activation)	TAU + SSRI	CES-D	HAM-D, CBC, YSR-112, SF-12, CGAS	Intervention had no effect on episodes of major depression
Courtney et al. (2022)	35	31	14–18	Depression	Outpatient psychiatric clinic United States	20 weeks	Integrated care pathway with standardised assessment, measurement-based care, a care giver intervention group and use of treatment protocol (CBT, medication algorithm, regular team reviews)	TAU	CDRS-R	WHO-DAS, CBC, CI-BPD, BHS	Decrease in CDRS-R across 4-week intervals in both groups. No significant effect of the intervention
Mufson et al. (2018)	29	19	12–18	Depression or dysthymia Excluded co-morbidities and active SI	Paediatric primary care United states	4 months	Co-location, use of evidence-based treatment protocol and stepped care (SCIPT-A: 8 weeks IPT, with a further 8 weeks if not recovered)	Enhanced TAU: referral within or outside the clinic, follow-up phone calls with SW to check on progress with referrals	CDRS-R	CGI-I	Intervention associated with non-significant increase in adherence to medication and greater reduction in symptoms
Richardson et al. (2014)	50	51	13–17	Depression Excluded co-morbidities and active SI	Paediatric primary care or family medicine clinics United states	12 months	Care coordination, family and carer involvement, use of treatment protocol (brief CBT ± medication)	Feedback on assessment, referral information	CDRS-R	–	Intervention associated with greater improvement in depressive symptoms
Shippee et al. (2018)	162	499	12–17	Depression or dysthymia Excluded co-morbidities and active SI	Paediatric primary care and family medicine United States	6 months	Care coordination, access to psychiatry consultation, use of evidence-based treatment protocol (behavioural activation, motivational interviewing)	PCP provided with a list of MH providers	PHQ-9-A	–	Intervention associated with greater remission and response
Staines et al. (2023)	188	389	13–15	Psychotic-like experiences	School-based Ireland	3 months	School-based screening, with supported MH referral for further assessment and intervention	Psychoeducation posters in classrooms	APSS	BDI, ZAS	Intervention associated with a significant reduction in symptoms relative to control arm
Weersing et al. (2017)	95	90	8–17	Depression, GAD, SAD, social phobia Excluded co-morbidities and active SI	Paediatric primary care United States	8–12 weekly sessions 4 months	Use of treatment protocol (brief behavioural therapy: exposure, behavioural activation, relaxation, problem solving, psychoeducation)	Limited care coordination (feedback on assessment, assisted referral to care, problem solving barriers to treatment, fortnightly contact)	CDRS-R	PARS, CGI-I, CGAS	Intervention associated with higher rates of clinical improvement, reduced symptoms and better functioning
Wuthrich et al. (2021)	32	21	12–18	Anxiety Excluded co-morbidities and active SI	Primary youth MH care Australia	8–10 weeks Up to 20 weeks	Stepped care intervention, combining digital and in-person structured psychological intervention supported by clinician phone calls	Up to 10 face-to-face sessions utilising various treatment modalities	CGI-severity	CGAS, CAS-8 or CHU9D	Intervention used significantly less therapy time, with similar benefits in clinical effectiveness, waiting time and QALYs

APSS: Adolescent Psychotic Symptom Screener; BDI: Beck Depression inventory; BHS: Beck Hopelessness Scale; CAS-8: Child Anxiety Scale-8; CBC: Child Behaviour Checklist; CBT: cognitive behavioural therapy; CES-D: Centre for Epidemiology Scale Depression; CDRS-R: Childhood Depression Rating Scale – Revised; CGI-I: Clinical Global Impression – Improvement; CGAS: Children’s Global Adjustment Scale; CHU9D: Child Health Utility 9D; CI-BPD: Childhood Inventory for Borderline Personality Disorder; GAD: Generalised Anxiety Disorder; HAM-D: Hamilton Depression scale; IPT: interpersonal therapy; MH: mental health; PARS: Paediatric Anxiety Rating Scale; PCP: primary care provider; PHQ-9: Patient Health Questionnaire-9; QALY: quality-adjusted life year; QoL: quality of life; SAD: separation anxiety disorder; SCIPT-A: stepped care interpersonal therapy – adolescent; SF-12: short form-12; SI: suicidal ideation; SSRI: selective serotonin reuptake inhibitor; SW: social worker; TAU: treatment as usual; WHO-DAS: World Health Organization – Disability Adjustment Scale; YMH: Youth mental health; YSR-112: Youth Self-Report-112; ZAS: Zung Anxiety Scale.

**Table 2.** Summary of studies evaluating impact of integrated YMH models on health service use.

Study	Participants, *n*		Sample and settling			Intervention			Outcome measures		
	Intervention	Control	Age (years)	Group	Setting	Timeframe of intervention	Intervention	Control group	Primary	Other	Significance
Rapp et al. (2017)	211	207	13–21	Depression	Primary care United States	6 months	Co-location, care coordination (CBT, medication + CBT)	PCP + additional training and materials	Access		Intervention associated with increased rates of access to care
Clarke et al. (2005)	75	77	12–18	Depression	Primary care United states	12 months	Co-location Use of treatment protocol (SSRI + brief CBT – cognitive restructuring or behavioural activation)	TAU (SSRI ± access to MH clinic)	Patient satisfaction	Access	Reduction in non-MH health visits and medications use, but not MH visits
Germán et al. (2017)	25,235	9417	6–18	All referred for MH assessment	Primary care clinics United states	6 months	Co-location, access to CAP, care coordination, use of treatment protocol (brief psychological intervention, PST)	TAU (generalist adult social worker)	Access	Provider satisfaction, provider self-reported competency	Increased referral rates, client and provider satisfaction and provider self-reported competency
Grimes et al. (2018)	32	196	12–18	All referred for MH assessment	Paediatric primary care clinics United States	6 weeks	Co-location, MDT with MH consultation model, weekly team meetings, shared treatment plans, eHR, care coordination, family peer workers	Referred to off-site child psychiatry	Access	Engagement (>1 follow-up activities, i.e. therapy, medication or other)	Intervention associated with four times greater OR of accessing care and seven times the OR of engaging in treatment
Lilly et al. (2020)	483	283	12–18	All referred for MH assessment	Primary care clinics United States	8 months	Co-location of MH providers	MH providers located in separate clinic	Access	Intake latency, cancellations	Intervention increased rates of clients scheduling initial appointments and decreased latency in first appointment. However, higher rates of cancellation and non-attendance
Mufson et al. (2018)	29	19	12–18	Depression or dysthymia Excluded co-morbidities and active SI	Paediatric primary care United states	4 months	Co-location, use of evidence-based treatment protocol and stepped care (SCIPT-A: 8 weeks IPT, with a further 8 weeks if not recovered)	Enhanced TAU: referral within or outside the clinic, follow up phone calls with SW to check on progress with referrals	Treatment completion	Attendance, adherence, patient, parent, provider satisfaction	SCIPT-A group reported increased adherence to medication, greater reduction in symptoms and improvement in illness severity. More access and less stigmatising care through the stepped care model of depression treatment.
Sterling et al. (2022)	1255	616	12–18	All referred for MH assessment, including substance use	Paediatric primary care United States	Single session (30–60 minutes)	SBIRT, PCPs referred to embedded BH clinician for further assessment, BI and referral for treatment as needed	Paediatricians had access to eHR screening tools but no formal SBIRT training	Healthcare visits (psychiatry, addiction medicine, ED, inpatient admissions)	MH diagnoses	Intervention associated with lower OR of substance use or alcohol diagnoses. MH diagnoses did not differ between the SBIRT and usual care groups
Wright et al. (2016)	50	51	13–17	Depression Excluded co-morbidities and active SI	Primary care clinics United States	12 months	Care coordination, evidence-based treatment protocol (psychotherapy ± medication)	Feedback on depression screening results	QALY, intervention cost, per capita health plan costs, cost-effectiveness ratio	CDRS-R	Found to be cost-effective compared to usual care

BH: behavioural health; BI: Brief intervention; CAP: child and adolescent psychiatrist; CBT: cognitive behavioural therapy; CDRS-R: Childhood Depression Rating Scale – Revised; CGI-I: Clinical Global Impression – Improvement; ED: Emergency department; eHR: electronic health record; IPT: interpersonal therapy; MDT: multidisciplinary team; MH: mental health; OR: odds ratio; PCP: primary care provider; PST: problem solving therapy; QALY: quality-adjusted life year; SBIRT: Screening, Brief Intervention and Referral to Treatment; SCIPT-A: stepped care interpersonal therapy – adolescent; SI: suicidal ideation; SSRI: selective serotonin reuptake inhibitor; SW: social worker; TAU: treatment as usual; YMH: Youth mental health.

Study quality was moderate to high in 11 studies, with scores of five on the Cochrane scale (see Supplemental Table 2). Four studies were of poor methodological rigour (score <3). Due to the nature of an integrated care intervention, true participant blinding was not feasible.

### Intervention characteristics

**Figure 3.**
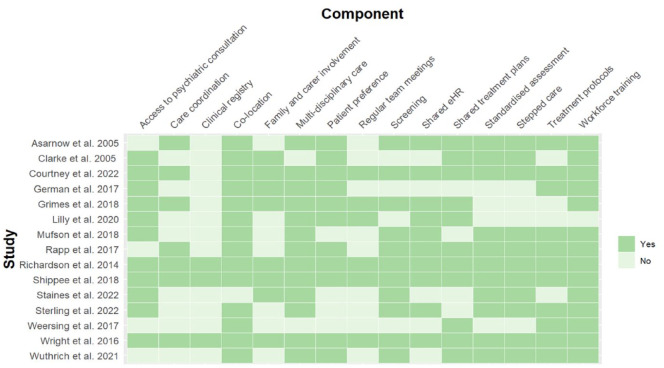
Integrated care components by study. eHR: electronic health record.

In all the studies, the mental health provider delivering the integrated intervention was co-located with a primary care provider (PCP). The integrated mental health intervention commonly involved a multidisciplinary team (13/15), with allocation of a care coordinator in half (7/15) of the studies (Figure 3). Seven of the 15 studies reported a psychiatric consultation model, rather than having a psychiatrist embedded within the team.

Other common components of the integrated intervention included use of shared treatment plans (11/15), shared electronic health records (eHRs; 10/15) and regular team meetings (6/15). The majority (11/15) of integrated care interventions used standardised treatment protocols or guidelines, which included brief structured psychological therapies or referred to medication algorithms. Workforce training in the integrated care intervention to be delivered was the most common feature across all studies (14/15). Use of screening tools in a primary care setting to ensure adequate case detection (11/15), standardised assessment using measurement-based care (11/15) and stepped care models (11/15) were less common.

Patient preference for a particular treatment pathway was included in the majority of integrated interventions (11/15); however, fewer studies included a component designed to engage family or carers (8/15). Of those studies that did include family engagement, this was generally limited to the initial engagement phase and focused on psychoeducation. Grimes et al. (2018) described family engagement in greater depth, which was delivered by family mentors with lived experience of caring for a family member with a mental health condition. Family mentors provided support in attending follow-up therapy sessions and in accessing information and resources.

The duration of intervention across studies ranged from 4 months to 1 year, with the median duration being 6 months. The frequency of therapy sessions varied from weekly to fortnightly. Richardson et al. (2014) reported the greatest frequency of contact between care coordinators and participants, with contact up to twice weekly, including via text message and phone.

TAU differed between studies, but in all studies follow-up care with a PCP was available. At a minimum, TAU involved feedback on assessment and the participants and PCPs being provided with a list of off-site mental health providers they could follow up with. TAU involved additional training for PCPs in two studies (Asarnow et al., 2005; Rapp et al., 2017), and an evidence-based medication protocol in another (Clarke et al., 2005). Two studies used an enhanced TAU procedure, which involved assistance with referrals and problem-solving barriers to referrals via fortnightly telephone follow-up with a mental health clinician (Mufson et al., 2018; Weersing et al., 2017).

### Clinical impact of integrated youth mental health models

Of the nine studies examining the impact of integration on clinical outcomes (Table 1), seven assessed the impact on depressive symptoms, and five of these studies also included a measure of anxiety (Weersing et al., 2017; Wuthrich et al., 2021) or other clinical symptoms via self-report (Asarnow et al., 2005; Clarke et al., 2005; Courtney et al., 2022; Staines et al., 2023). Three studies measured global clinical improvement (Clarke et al., 2005; Mufson et al., 2018; Weersing et al., 2017), two measured level of functioning (Asarnow et al., 2005; Clarke et al., 2005) and only one study measured quality of life (Courtney et al., 2022). Six of the seven studies reported a greater improvement in depressive symptoms in the intervention group, with increased rates of remission at follow-up compared to TAU. The integrated care intervention was associated with improved functioning relative to TAU in all seven studies. Depressive symptoms were the only outcome measured across a sufficient number of studies to enter into meta-analysis.

### Health service outcomes of integrated youth mental health models

Of the seven studies examining the impact of integration on health service use, five measured rates of access or attendance (Germán et al., 2017; Grimes et al., 2018; Mufson et al., 2018; Rapp et al., 2017; Sterling et al., 2022), and three measured engagement beyond the initial assessment (Grimes et al., 2018; Lilly et al., 2020). All studies reported increased rates of access and engagement in with mental health care in the intervention group. Sterling et al. (2022) also reported a reduction in hospital-based health visits in the intervention group. Grimes et al. (2018) compared a co-located mental health intervention, that included care coordination, regular team meetings, shared treatment planning and eHR and family involvement to TAU (referrals to off-site child psychiatry) and found those in the intervention group were four times more likely to access care and seven times more likely to engage in treatment, including psychotherapy or medication, than controls (Grimes et al., 2018).

Three studies evaluated patient satisfaction and two studies also measured provider satisfaction (Clarke et al., 2005; Germán et al., 2017). Asarnow et al. (2005) reported that the integrated intervention was associated with significantly higher patient treatment satisfaction. However, two other studies reported no difference in satisfaction between the groups, with both reporting high levels of satisfaction (Clarke et al., 2005; Mufson et al., 2018). The two studies that assessed an integrated care model, characterised by co-location of a mental health clinician and use of standardised assessment and treatment protocol, found differing results for provider satisfaction. Germán et al. (2017) reported social workers and primary care physicians were more satisfied with a number of aspects of the intervention, including the ease of referring, reduced wait times, feedback received from mental health clinicians and improved care for their patients. There was no effect on the provider’s self-reported competence in treating mental health conditions. Mufson et al. (2018) reported no significant differences in provider satisfaction between intervention and control arms.

### Meta-analysis

**Figure 4.**
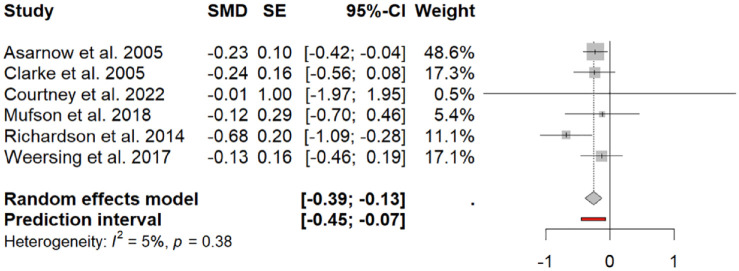
Forest plot of effect size as difference in depression severity association with integrated intervention at 4- or 6-month follow-up. SMD: standardised mean difference; SE: standard error; CI: confidence interval.

The meta-analysis included six studies, which assessed the impact of integration on depressive symptoms, which included a total of 970 participants (475 in intervention, 495 controls). All studies included at least one time-point that assessed depressive symptoms at 4–6 months of follow-up. Pooled effect size found that the integrated intervention was associated with a significantly greater reduction in depressive symptoms relative to controls at 4–6 months, with a small effect size (SMD = −0.260, 95% CI = [−0.39, −0.13], *p* = 0.001; Figure 4). There was no significant difference between studies regarding heterogeneity (*Q*-statistics = 5.26, df (*Q*) = 5, *p* = 0.38, *I*^2^ = 5%). An Egger’s regression was non-significant (intercept −0.25, 95% CI = [−0.56, 0.06], *p* = 0.92).

Given the study by Richardson et al. (2014) reported a much larger effect size than other studies, a sensitivity analysis was conducted with the study removed. The pooled effect size remained significant (SMD = −0.207, 95% CI = [−0.35, −0.07], *p* = 0.0042).

## Discussion

Integrated models of mental healthcare are associated with greater effectiveness in reduction of clinical symptoms than non-integrated models of care (TAU). Although our meta-analysis was limited by the small number of studies and heterogeneity in outcome measures, a small, but significant, effect of integrated care on clinical symptoms was apparent. These findings are consistent with a previous meta-analysis that reported a small to medium sized effect of integrated care on behavioural symptoms in studies of children and adolescents (Asarnow et al., 2015). Our findings confirm that integrated care is associated with a small increase in clinical effectiveness of mental healthcare in an older cohort (12–25 years). Integrated care was also associated with increased rates of access to mental health interventions in primary care settings and, in a number of studies, with increased engagement beyond initial contact. While the majority of studies focused on depressive or anxiety symptoms and excluded common markers of complexity, such as co-morbid diagnoses or suicidality, the increased rates of engagement and treatment completion in participants receiving an integrated intervention were encouraging and may indicate that young people with complex mental health presentations may stand to benefit from integrated care.

Although models of integration varied between studies, some components were central to the delivery of an integrated care intervention. Shared treatment plans and use of treatment protocols, which involved clinicians receiving specific training and supervision in the model, were the most frequent components across models. Care coordination was another component central to more complex interventions where there were a greater number of components to integrate. Care coordinators, or care managers (as they are referred to in the United States), facilitated greater access to information and advocated for the client’s and/or family or carer’s preferences.

In most interventions, care coordinators were involved in assessment and treatment planning, particularly where standardised assessment measures and treatment protocols and stepped care algorithms were used. In a number of studies, it was coordinators who delivered structured psychological interventions according to a treatment guideline, such as psychoeducation or behavioural activation (Björkelund et al., 2018; Shippee et al., 2018; Wright et al., 2016). Care coordinators also assisted with advocacy and contact with outside agencies (e.g. school, social services), as well as any transitions between services and clinicians (Yonek et al., 2020).

Co-location was a feature of integration in all the reviewed studies; however, how exactly this impacted collaboration between providers and service delivery was not always explicit. Less than half of interventions stipulated that regular clinical team meetings for case review or care planning occurred. Co-location has previously been described as increasing the ease of clinicians making a referral and receiving feedback as well as seeking informal advice from colleagues (Walton et al., 2022). It is important to note that all studies in the review were published prior to the COVID-19 pandemic. The role of physical co-location in delivering integrated mental health care should be reconsidered in light of the paradigm shift in service delivery that has occurred with the pandemic. The reviewed studies primarily used in-person forms of communication with clients, although some follow-up by telephone was common between sessions. A hybrid of in-person and remote contact was more common in communication between providers, with two thirds of studies reporting communication via phone and use of shared eHR. However, use of digital platforms for other aspects of service delivery, such as clinical review meetings or collecting assessment measures, was not described.

In post-COVID-19 pandemic health systems, digital platforms are routinely used for all aspects of service delivery, such as assessment and triage, referrals, treatment planning and collaboration within teams and with external providers. Considering these changes to service delivery, the impact of co-location on integration of service delivery (i.e. increased ease of communication or referral processes between clinicians) may be lessened. Similarly, the adoption of digital platforms for collaboration within clinical teams and with external providers and the continuation of hybrid in-office and remote working for much of the workforce are likely to impact the effect of physical co-location.

Despite the increased digital connectivity between providers, it is notable that some key components of integration, such as access to a shared eHR, have not yet been implemented widely in Australia and many other health systems. In Australia, progress is being made on a single digital health record and increasing interoperability between clinical systems that would allow for greater collaboration between providers (Australian Digital Health Agency, 2016; NSW Government, 2016). Similarly, many providers, including federal and state governments, have been developing digital platforms to collect patient-reported outcomes (NSW Government, 2022; Peterson et al., 2019), which is likely to facilitate the use of standardised assessment and outcome measures in mental healthcare and may improve the quality of data collection within services. For the individual consumer, streamlining the collection of information required in clinical assessments can reduce the burden on consumers by reducing duplication and improve the quality of information shared between providers. Incorporating digital data collection into service delivery can also increase the feasibility of services maintaining clinical registries, which inform system-level health planning and the monitoring and evaluation required to maintain integration.

There are a number of limitations to consider in the current review. First, system-level components of integration were not included in any study. E.g. the design of governance structures, the development of intersectoral partnerships and shared funding models are all considered key enablers of integrated care (12). It remains a challenge to assess the impact of these macro-level factors, which are often complex and dynamic, on downstream clinical outcomes and experience of care. However, the inclusion of these macro-level factors would provide important context when assessing the impact of integrated care interventions at a service and individual level. Given the majority of studies were set in the United States, where macro-level factors may differ markedly from other health systems, caution is required in generalising these results to other contexts.

It should also be noted that there was a high level of satisfaction with TAU in the control groups. A possible explanation for this is that the nature of many research study designs means that some components of integrated care may be inherent to a study protocol and thus delivered to participants in both the intervention and control arm. E.g. the control arm in several studies included standardised assessment and feedback on outcome, followed by communication to a PCP and, in some studies, support with onward referrals. In real-world settings, TAU can vary widely between and within countries, such as between urban and rural areas. The standard of clinical care is also known to vary between services within the same city due to different factors, such as workforce or funding shortages. As a result, the current findings may underestimate the positive impact integrated care models may have in some settings.

Other limitations of the current review include the small number of studies and the exclusion of young people with co-morbidities, such as suicidal behaviour or substance use, from many studies. These features are common in young people accessing mental health services (Filia et al., 2021) and such exclusions may limit the generalisability of the findings to clinical samples with even a moderate degree of complexity. Given integration can increase access and engagement with care, future research should assess the impact of integration on complex mental health conditions, including trait-based symptom clusters associated with chronicity.

The implications for service development, keeping in mind these limitations, are that our review supports the implementation of integrated care models for mental healthcare for adolescents and young adults, consistent with findings for children and adolescents. Integrated care provides improved access and engagement, better clinical outcomes and high service satisfaction for young people. The cost-effectiveness of integrated care compared to TAU needs to be established, as integration entails additional components, including training and supervision, care coordinator roles and possibly co-location, although the latter needs to be further investigated in the post-COVID environment. Similarly, the role of technology in integration requires further examination, as this is likely to be a cost-effective way to further enhance the healthcare integration. Given the central role of workforce factors in the sustainability of health service models, further understanding of provider satisfaction and competency in integrated services relative to TAU is also needed. Nevertheless, the value of integration to improved engagement and outcomes for young people is likely to warrant the investment. Further research and evaluation of the impact of integration of mental health care with a broader range of services and settings are also needed, e.g. understanding the impact of integrating youth mental health care with First Nations health services or with social services, such as education or housing.

## Conclusion

Integrated models of mental healthcare are associated with a small, but significant, increase in clinical effectiveness for depressive symptoms relative to TAU. Given integrated care models may increase access and engagement, future research should also focus on assessing the impact of integrated care for young people with complex mental health presentations including suicidality and co-morbid conditions, including clinical and functional recovery, satisfaction with care as well as health system outcomes such as cost-effectiveness and workforce satisfaction.

## Supplemental Material

sj-docx-1-anp-10.1177_00048674241256759 – Supplemental material for Integrated care models for youth mental health: A systematic review and meta-analysis Supplemental material, sj-docx-1-anp-10.1177_00048674241256759 for Integrated care models for youth mental health: A systematic review and meta-analysis by Catherine McHugh, Nan Hu, Gabrielle Georgiou, Michael Hodgins, Sarah Leung, Mariyam Cadiri, Nicola Paul, Vikki Ryall, Debra Rickwood, Valsamma Eapen, Jackie Curtis and Raghu Lingam in Australian & New Zealand Journal of Psychiatry

## References

[bibr1-00048674241256759] Archer J Bower P Gilbody S , et al. (2012) Collaborative care for depression and anxiety problems. Cochrane Database of Systematic Reviews 10: CD006525. 10.1002/14651858.CD006525.pub2 PMC11627142 23076925

[bibr2-00048674241256759] Asarnow JR Jaycox LH Duan N , et al. (2005) Effectiveness of a quality improvement intervention for adolescent depression in primary care clinics: A randomized controlled trial. JAMA 293: 311–319. 15657324 10.1001/jama.293.3.311

[bibr3-00048674241256759] Asarnow JR Rozenman M Wiblin J , et al. (2015) Integrated medical-behavioral care compared with usual primary care for child and adolescent behavioral health: A meta-analysis. JAMA Pediatrics 169: 929–937. 26259143 10.1001/jamapediatrics.2015.1141

[bibr4-00048674241256759] Australian Digital Health Agency (2016) National Digital Health Strategy and Framework for Action: Safe, Seamless and Secure: Evolving Health and Care to Meet the Needs of Modern Australia. Sydney, NSW, Australia: Australian Digital Health Agency.

[bibr5-00048674241256759] Bartholomeusz C Randell A (2022) Defining integrated care and its core components in youth mental health. Part 3: Findings from consultations with key stakeholders. Available at: www.orygen.org.au/Training/Resources/integrated-care-models/Findings-from-consultations-with-key-stakeholders (accessed 15 May 2024).

[bibr6-00048674241256759] Björkelund C Svenningsson I Hange D , et al. (2018) Clinical effectiveness of care managers in collaborative care for patients with depression in Swedish primary health care: A pragmatic cluster randomized controlled trial. BMC Family Practice 19: 28. 29426288 10.1186/s12875-018-0711-z PMC5807835

[bibr7-00048674241256759] Burkhart K Asogwa K Muzaffar N , et al. (2020) Pediatric integrated care models: A systematic review. Clinical Pediatrics 59: 148–153. 31762297 10.1177/0009922819890004

[bibr8-00048674241256759] Clarke G Debar L Lynch F , et al. (2005) A randomized effectiveness trial of brief cognitive-behavioral therapy for depressed adolescents receiving antidepressant medication. Journal of the American Academy of Child and Adolescent Psychiatry 44: 888–898. 16113617

[bibr9-00048674241256759] Courtney DB Cheung A Henderson J , et al. (2022) CARIBOU-1: A pilot controlled trial of an Integrated Care Pathway for the treatment of depression in adolescents. JCPP Advances 2: e12083. 10.1002/jcv2.12083 PMC10242836 37431464

[bibr10-00048674241256759] Egger M Smith GD Schneider M , et al. (1997) Bias in meta-analysis detected by a simple, graphical test. BMJ 315: 629–634. 9310563 10.1136/bmj.315.7109.629 PMC2127453

[bibr11-00048674241256759] Filia K Rickwood D Menssink J , et al. (2021) Clinical and functional characteristics of a subsample of young people presenting for primary mental healthcare at headspace services across Australia. Social Psychiatry and Psychiatric Epidemiology 56: 1311–1323. 33452888 10.1007/s00127-020-02020-6

[bibr12-00048674241256759] Fusar-Poli P (2019) Integrated mental health services for the developmental period (0 to 25 years): A critical review of the evidence. Frontiers in Psychiatry 10: 355. 31231250 10.3389/fpsyt.2019.00355 PMC6567858

[bibr13-00048674241256759] Fusar-Poli P McGorry PD Kane JM (2017) Improving outcomes of first-episode psychosis: An overview. World Psychiatry 16: 251–265. 28941089 10.1002/wps.20446 PMC5608829

[bibr14-00048674241256759] Germán M Rinke ML Gurney BA , et al. (2017) Comparing two models of integrated behavioral health programs in pediatric primary care. Child and Adolescent Psychiatric Clinics of North America 26: 815–828. 28916016 10.1016/j.chc.2017.06.009

[bibr15-00048674241256759] Grimes KE Creedon TB Webster CR , et al. (2018) Enhanced child psychiatry access and engagement via integrated care: A collaborative practice model with pediatrics. Psychiatric Services 69: 986–992. 30041586 10.1176/appi.ps.201600228

[bibr16-00048674241256759] Henry LP Amminger GP Harris MG , et al. (2010) The EPPIC follow-up study of first-episode psychosis: Longer-term clinical and functional outcome 7 years after index admission. The Journal of Clinical Psychiatry 71: 716–728. 20573330 10.4088/JCP.08m04846yel

[bibr17-00048674241256759] Hetrick SE Bailey AP Smith KE , et al. (2017) Integrated (one-stop shop) youth health care: Best available evidence and future directions. Medical Journal of Australia 207: S5–S18. 10.5694/mja17.00694 29129182

[bibr18-00048674241256759] Higgins JPT Altman DG Gøtzsche PC , et al. (2011) The Cochrane Collaboration’s tool for assessing risk of bias in randomised trials. BMJ 343: d5928. 10.1136/bmj.d5928 PMC3196245 22008217

[bibr19-00048674241256759] Hodgins M McHugh C Hu N , et al. (2022) Review of integrated care in youth mental health. https://headspace.org.au/assets/Review-of-integrated-care-in-youth-mental-health-FINAL.pdf (accessed 15 May 2024).

[bibr20-00048674241256759] Holst A Ginter A Björkelund C , et al. (2018) Cost-effectiveness of a care manager collaborative care programme for patients with depression in primary care: Economic evaluation of a pragmatic randomised controlled study. BMJ Open 8: e024741. 10.1136/bmjopen-2018-024741 PMC6252772 30420353

[bibr21-00048674241256759] Hu N Nassar N Shrapnel J , et al. (2022) The impact of the COVID-19 pandemic on paediatric health service use within one year after the first pandemic outbreak in New South Wales Australia – A time series analysis. The Lancet Regional Health – Western Pacific 19: 100311. 34746898 10.1016/j.lanwpc.2021.100311 PMC8564784

[bibr22-00048674241256759] Kalb LG Stapp EK Ballard ED , et al. (2019) Trends in psychiatric emergency department visits among youth and young adults in the US. Pediatrics 143: e20182192. 10.1542/peds.2018-2192 PMC6564072 30886112

[bibr23-00048674241256759] Lilly RG Meadows TJ Sevecke-Hanrahan JR , et al. (2020) Hub-extension model and access to pediatric behavioral integrated primary care. Clinical Practice in Pediatric Psychology 8: 220.

[bibr24-00048674241256759] Malla A Iyer S McGorry P , et al. (2016) From early intervention in psychosis to youth mental health reform: A review of the evolution and transformation of mental health services for young people. Social Psychiatry and Psychiatric Epidemiology 51: 319–326. 26687237 10.1007/s00127-015-1165-4

[bibr25-00048674241256759] Mercado MC Holland K Leemis RW , et al. (2017) Trends in emergency department visits for nonfatal self-inflicted injuries among youth aged 10 to 24 years in the United States, 2001-2015. JAMA 318: 1931–1933. 29164246 10.1001/jama.2017.13317 PMC5753998

[bibr26-00048674241256759] Moher D Liberati A Tetzlaff J , et al. (2009) Preferred reporting items for systematic reviews and meta-analyses: The PRISMA statement. Annals of Internal Medicine 151: 264–269. 19622511 10.7326/0003-4819-151-4-200908180-00135

[bibr27-00048674241256759] Mufson L Rynn M Yanes-Lukin P , et al. (2018) Stepped care interpersonal psychotherapy treatment for depressed adolescents: A pilot study in pediatric clinics. Administration and Policy in Mental Health 45: 417–431. 29124527 10.1007/s10488-017-0836-8 PMC5911397

[bibr28-00048674241256759] NSW Government (2016) eHealth Strategy for NSW Health 2016-2026: A digitally enabled and integrated health system delivering patient-centred health experiences and quality health outcomes. www.health.nsw.gov.au/priorities/documents/ehealth-strategy.pdf (accessed 15 May 2024).

[bibr29-00048674241256759] NSW Government (2022) Health Outcomes and Patient Experience (HOPE) platform. Available at: https://aci.health.nsw.gov.au/statewide-programs/prms/hope-platform (accessed 15 May 2024).

[bibr30-00048674241256759] Perera J Wand T Bein KJ , et al. (2018) Presentations to NSW emergency departments with self-harm, suicidal ideation, or intentional poisoning, 2010–2014. Medical Journal of Australia 208: 348–353. 29669496 10.5694/mja17.00589

[bibr31-00048674241256759] Peterson K Anderson J Bourne D (2019) Evidence Brief: Use of Patient Reported Outcome Measures for Measurement Based Care in Mental Health Shared Decision-Making. Washington, DC: Department of Veterans Affairs. 30645065

[bibr32-00048674241256759] Rapp AM Chavira DA Sugar CA , et al. (2017) Integrated primary medical-behavioral health care for adolescent and young adult depression: Predictors of service use in the youth partners in care trial. Journal of Pediatric Psychology 42: 1051–1064. 28369443 10.1093/jpepsy/jsx057 PMC5896616

[bibr33-00048674241256759] Reardon T Harvey K Baranowska M , et al. (2017) What do parents perceive are the barriers and facilitators to accessing psychological treatment for mental health problems in children and adolescents? A systematic review of qualitative and quantitative studies. European Child & Adolescent Psychiatry 26: 623–647. 28054223 10.1007/s00787-016-0930-6 PMC5446558

[bibr34-00048674241256759] Richards DA Bower P Chew-Graham C , et al. (2016) Clinical effectiveness and cost-effectiveness of collaborative care for depression in UK primary care (CADET): A cluster randomised controlled trial. Health Technology Assessment (Winchester, England) 20: 1–192. 10.3310/hta20140 PMC4809468 26910256

[bibr35-00048674241256759] Richardson LP Ludman E McCauley E , et al. (2014) Collaborative care for adolescents with depression in primary care: A randomized clinical trial. JAMA 312: 809–816. 25157724 10.1001/jama.2014.9259 PMC4492537

[bibr36-00048674241256759] Rickwood D Nicholas A Mazzer K , et al. (2017) Satisfaction with youth mental health services: Further scale development and findings from headspace–Australia’s National Youth Mental Health Foundation. Early Intervention in Psychiatry 11: 296–305. 25996832 10.1111/eip.12248

[bibr37-00048674241256759] Rickwood D Paraskakis M Quin D , et al. (2019) Australia’s innovation in youth mental health care: The headspace centre model. Early Intervention in Psychiatry 13: 159–166. 30311423 10.1111/eip.12740 PMC6585724

[bibr38-00048674241256759] Rickwood DJ Mazzer KR Telford NR , et al. (2015) Changes in psychological distress and psychosocial functioning in young people visiting headspace centres for mental health problems. Medical Journal of Australia 202: 537–542. 26021366 10.5694/mja14.01696

[bibr39-00048674241256759] Shippee ND Mattson A Brennan R , et al. (2018) Effectiveness in regular practice of collaborative care for depression among adolescents: A retrospective cohort study. Psychiatric Services 69: 536–541. 29446330 10.1176/appi.ps.201700298

[bibr40-00048674241256759] Staines L Healy C Corcoran P , et al. (2023) Investigating the effectiveness of three school based interventions for preventing psychotic experiences over a year period – a secondary data analysis study of a randomized control trial. BMC Public Health 23: 219. 36726107 10.1186/s12889-023-15107-x PMC9890687

[bibr41-00048674241256759] State of Victoria (2021) Royal Commission into Victoria’s Mental Health System: Final report. Parl Paper No. 202, Session 2018–2021.

[bibr42-00048674241256759] Sterling S Parthasarathy S Jones A , et al. (2022) Young adult substance use and healthcare use associated with screening, brief intervention and referral to treatment in pediatric primary care. The Journal of Adolescent Health 71: S15–S23. 10.1016/j.jadohealth.2021.11.033 36122965

[bibr43-00048674241256759] Walton QL Bromley E Porras-Javier L , et al. (2022) Building bridges: Primary care and mental health providers’ perspectives on a behavioral health collaborative intervention among underserved populations. Child & Youth Care Forum 51: 495–514.

[bibr44-00048674241256759] Weersing VR Brent DA Rozenman MS , et al. (2017) Brief behavioral therapy for pediatric anxiety and depression in primary care: A randomized clinical trial. JAMA Psychiatry 74: 571–578. 28423145 10.1001/jamapsychiatry.2017.0429 PMC5539834

[bibr45-00048674241256759] Western Australian Association for Mental Health (2018) Report on the Youth Mental Health Services Integration Project. Perth, WA, Australia: WAAMH.

[bibr46-00048674241256759] World Health Organization (2016a) Integrated care models: An overview. Available at: https://wyoleg.gov/InterimCommittee/2019/10-201906132.WHOIntegrated-care-models-overview.pdf (accessed 15 May 2024).

[bibr47-00048674241256759] World Health Organization (2016b) Strengthening integrated, people-centred health services. Available at: https://iris.who.int/bitstream/handle/10665/252804/A69_R24-en.pdf?sequence=1 (accessed 15 May 2024).

[bibr48-00048674241256759] Wright DR Haaland WL Ludman E , et al. (2016) The costs and cost-effectiveness of collaborative care for adolescents with depression in primary care settings: A randomized clinical trial. JAMA Pediatrics 170: 1048–1054. 27654449 10.1001/jamapediatrics.2016.1721

[bibr49-00048674241256759] Wuthrich VM Rapee RM McLellan L , et al. (2021) Psychological stepped care for anxious adolescents in community mental health services: A pilot effectiveness trial. Psychiatry Research 303: 114066. 34175714 10.1016/j.psychres.2021.114066

[bibr50-00048674241256759] Yonek J Lee C-M Harrison A , et al. (2020) Key components of effective pediatric integrated mental health care models: A systematic review. JAMA Pediatrics 174: 487–498. 32150257 10.1001/jamapediatrics.2020.0023 PMC7483725

