# Supplementary material for: Integrated care models for youth mental health: A systematic review and meta-analysis
Source: Aust N Z J Psychiatry. 2024 Jun 7;58(9):747–59. doi: 10.1177/00048674241256759 (PMC11370150; doi:10.1177/00048674241256759)
Supplement: sj-docx-1-anp-10.1177_00048674241256759 – Supplemental material for Integrated care models for youth mental health: A systematic review and meta-analysis [file sj-docx-1-anp-10.1177_00048674241256759.docx]

**Supplementary Table 1.**

| **Children and young people group:**  pediatric* OR paediatric* OR teen* OR adolescen* OR pubescent OR “young people” OR youth* OR pubert* OR “young adult” |
| --- |
| **Mental health condition:**  “mental health*” OR “mental disorders” OR “mental health services*” |
| **Integrated care model:**  “integrat*” OR “integrated care” OR “colocat*” OR “care coordination” OR “collaborative care” OR “coordinated care” OR “horizontal integration” OR “vertical integration” OR “longitudinal integration” OR “virtual integration” OR “medical home” OR "health services*" OR "delivery of health care*". |

**Supplementary table 2.** Risk of bias assessment of included studies

| Study | Design | Random sequence generation | Allocation concealment | Blinding of participants and personnel | Blinding of outcome assessment | Incomplete outcome data | Selective reporting |
| --- | --- | --- | --- | --- | --- | --- | --- |
| Asarnow et al 2005 | RCT |  | 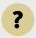 | 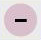 |  |  |  |
| Clarke et al 2005 | RCT | 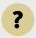 | 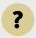 | 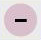 |  |  |  |
| Courtney et al. 2022 | RCT | 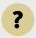 | 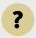 | 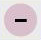 |  |  |  |
| Mufson et al 2018 | RCT |  | 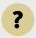 | 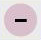 |  |  |  |
| Richardson et al 2014 | RCT |  | 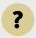 | 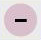 |  |  |  |
| Weersing et al 2017 | RCT |  | 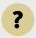 | 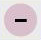 |  |  |  |

- Low risk of bias;
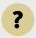
 Unclear risk of bias;
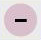
 High risk of bias
